# Non-Destructive Structural Identification of Camouflaged Microcavity Displays via Digital Twin-Assisted Electroluminescence Analysis

**DOI:** 10.3390/nano16181128

**Published:** 2026-09-09

**Authors:** Ming-Yi Lin, Cheng-Hao Cheng, Shu-Han Wu, Chun-Ying Huang, Cheng-Yuan Chang

**Affiliations:** 1 Department of Electrical Engineering, National United University, Miaoli 36003, Taiwan; mylin@nuu.edu.tw (M.-Y.L.); bob30610@gmail.com (C.-H.C.); qwer2623260@gmail.com (S.-H.W.); 2 Department of Applied Materials and Optoelectronic Engineering, National Chi Nan University, Nantou 54561, Taiwan

**Keywords:** microcavity displays, physics-informed machine learning, spectral camouflage, digital twin-assisted modeling, non-destructive structural identification

## Abstract

In microcavity displays, different device structures can produce nearly identical normal-incidence electroluminescence spectra, making non-destructive identification difficult. This is especially relevant when narrow-band QLED emission is compared with cavity-narrowed OLED emission. Angle-resolved measurements can distinguish these cases, but the need for mechanical rotation limits measurement throughput. Here, we developed a digital twin-assisted method that uses a single normal-incidence spectrum for structural identification. The optical model was parameterized with measured material properties and checked against measured electroluminescence spectra. It was then used to generate spectra with ±1 nm electrode-thickness variations, and measured spectra were also included during training. Four machine-learning classifiers were compared for eight QLED/OLED device structures. The Tanh-activated multilayer perceptron gave the highest testing accuracy of 93.94%, compared with 84.85% for logistic regression. These results show that small differences in the full spectral shape can support structural identification when peak wavelength and linewidth alone are ambiguous. The method provides a practical basis for rotation-free optical screening of microcavity display structures.

## 1. Introduction

The increasing use of Quantum Dot Light-Emitting Diodes (QLEDs) and Organic Light-Emitting Diodes (OLEDs) in high-end displays has created a need for reliable non-destructive inspection and structural verification [1,2,3]. In manufacturing and procurement, devices with different internal structures can have similar external appearances and similar emission characteristics, which makes rapid identification difficult when many components must be screened. Statistical sampling alone may miss infrequent defective, out-of-specification, or incorrectly mixed components. High-throughput in-line optical inspection is therefore useful for display quality control.

A major difficulty is the spectral similarity between intrinsically narrow-band QLED emission and cavity-narrowed OLED emission. QLEDs typically show narrow electroluminescence spectra because of the size-dependent emission of quantum dots. OLEDs can also produce narrow normal-incidence spectra when reflective electrodes form a strong microcavity [4,5,6]. Under these conditions, devices with different structures may have very similar electroluminescence spectra at normal incidence. We refer to this condition as spectral camouflage. In such cases, common descriptors such as peak wavelength and full width at half maximum (FWHM) may not be sufficient for reliable structural identification.

Angle-resolved measurements can distinguish these cases because microcavity emission changes with viewing angle [7,8]. In microcavity OLEDs, the resonance wavelength usually shifts with angle, providing a useful signature of cavity strength. However, goniometric measurements require mechanical rotation, optical alignment, and repeated spectral acquisition at several angles. These steps reduce throughput and make angle-resolved measurements less suitable for high-volume in-line inspection [9].

Machine learning has been increasingly used to analyze optical spectra for inspection and classification [10,11,12]. Its application to structural identification, however, depends on having training data that represent realistic device variations. Fabricating large numbers of QLED and OLED samples with controlled thickness errors and different cavity strengths is costly and time-consuming. In addition, classifiers based only on selected descriptors such as peak wavelength and FWHM may fail when spectra from different structures overlap strongly [13,14,15]. For this reason, the full electroluminescence spectrum is considered here together with device variations derived from the optical model.

**Figure 1:**
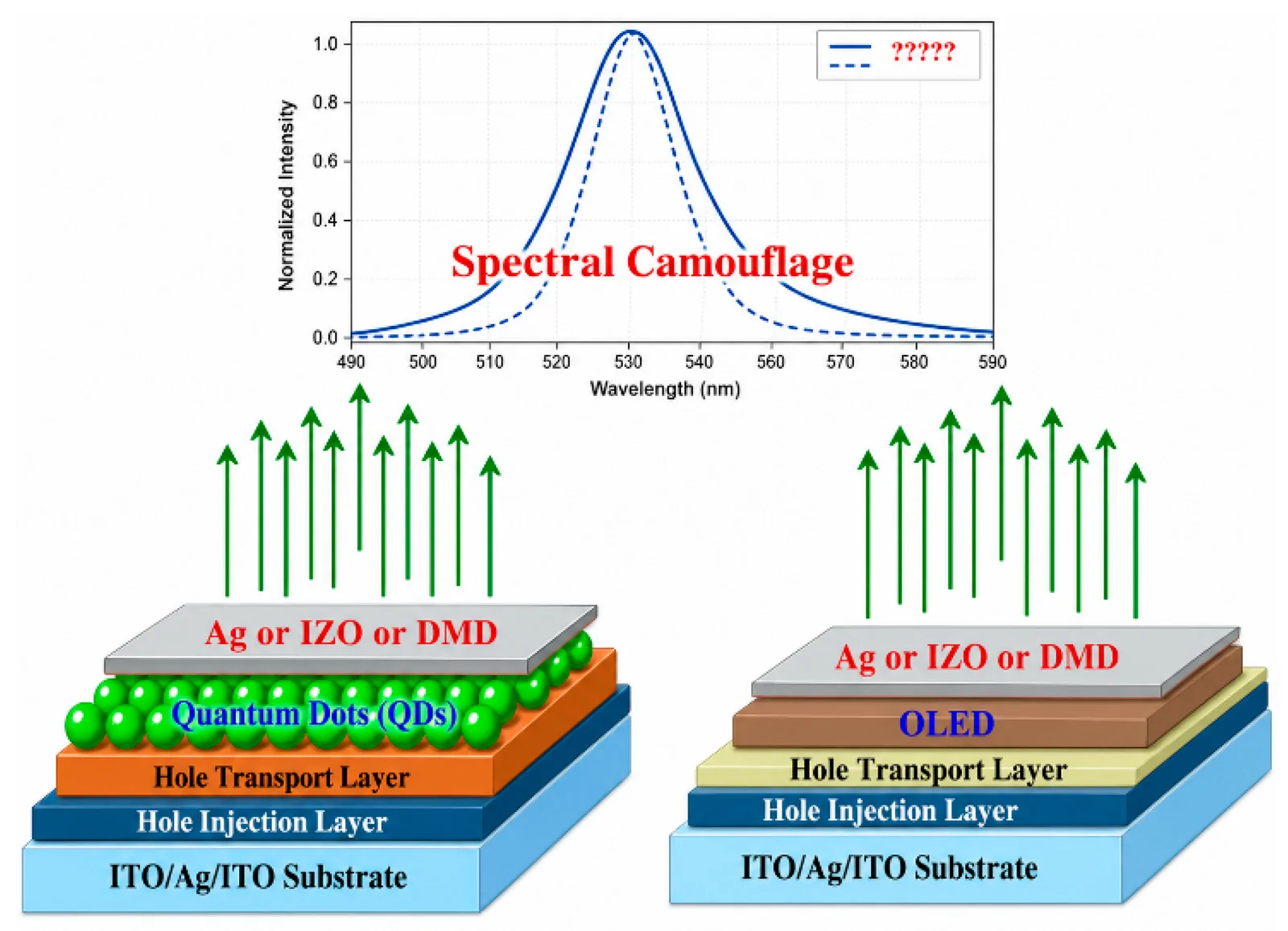
Spectral camouflage in QLED and OLED microcavity displays. Structurally different devices can exhibit similar normal-incidence electroluminescence spectra, motivating non-destructive structural identification. Green arrows schematically indicate emitted light, while the layer colors are used for visual distinction.

In this work, we combine an experimentally parameterized optical digital twin with machine-learning classification to identify spectrally camouflaged microcavity displays from normal-incidence electroluminescence spectra (Figure 1). The digital twin was used to generate spectra that include ±1 nm electrode-thickness variations, reducing the need to fabricate a large number of physical samples. Four classifiers—Gradient Descent, support vector machine (SVM), Logistic Regression, and a Tanh-activated multilayer perceptron (MLP)—were evaluated. This comparison was used to examine whether the nonlinear MLP provides an advantage over linear and kernel-based methods when the spectra overlap strongly. The approach is intended as a single-shot screening method for QLED and OLED microcavity structures without mechanical angle-resolved measurements.

## 2. Experimental Details

Before constructing the digital twin, we established an experimental optical baseline for the emissive quantum-dot layer. The measured optical properties were then used as inputs to the subsequent simulations.

**Figure 2:**
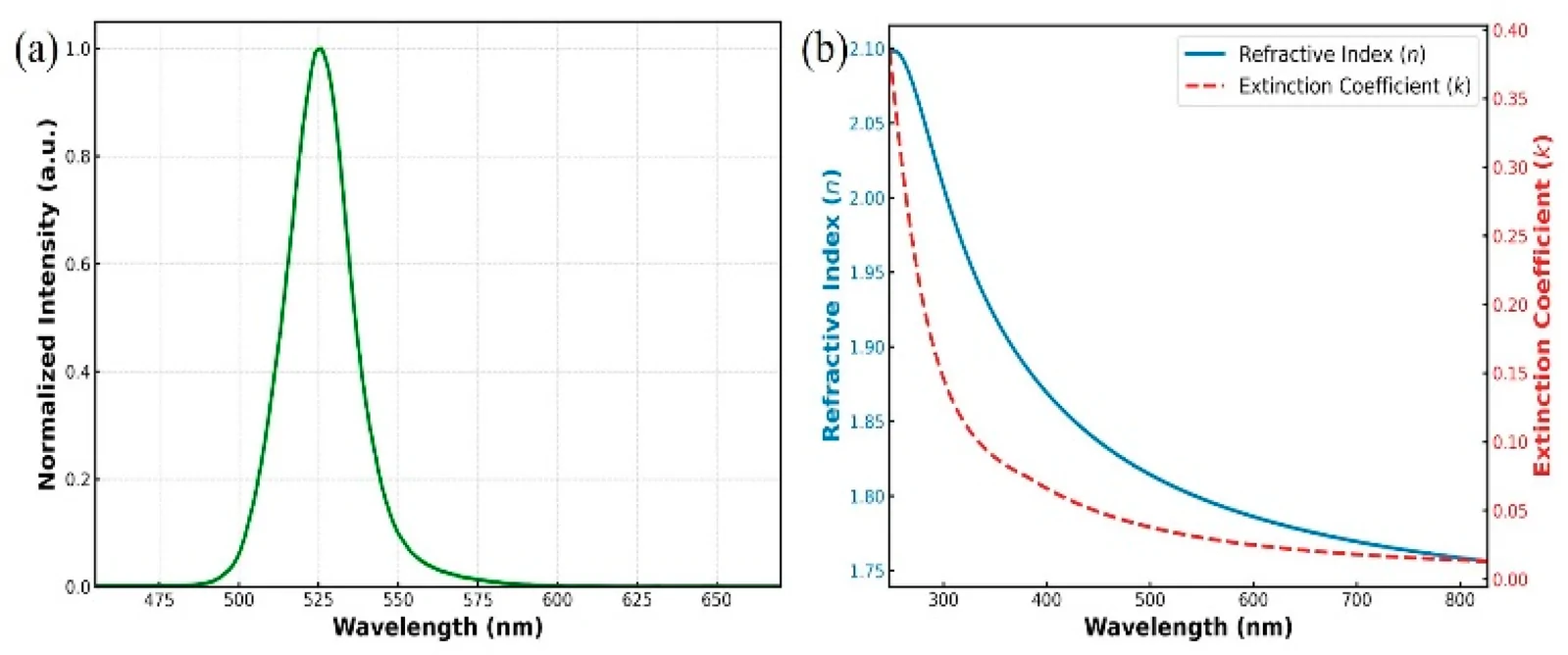
Experimental optical parameterization of the green quantum-dot layer. (**a**) Measured photoluminescence spectrum of the GQDs. (**b**) Measured wavelength-dependent refractive index (n) and extinction coefficient (k), used as input material parameters for digital twin-assisted optical simulations.

Figure 2a shows the measured photoluminescence (PL) spectrum of the green quantum dots (GQDs). The narrow green emission band was used as the intrinsic emission profile before microcavity modulation. Figure 2b shows the measured wavelength-dependent complex refractive index of the GQD layer. The refractive index (n) and extinction coefficient (k) determine phase propagation and optical loss and are needed to calculate interference, absorption, and cavity-induced changes in the multilayer emission spectrum.

The measured PL spectrum and n/k dispersion were used directly in the optical model. In this way, the simulated electroluminescence spectra were based on measured material properties rather than idealized optical inputs, providing the basis for generating the spectral datasets used for classification.

The optical model was implemented in Fluxim Setfos^®^. The simulated top-emitting devices used a 125 nm Ag bottom electrode, a 20 nm hole-injection layer (HIL), and a 25 nm hole-transport layer (HTL). The QLED structure contained a 14 nm quantum-dot emissive layer, whereas the OLED structure used a 39 nm organic emissive layer. Four top-electrode configurations were considered: thin Ag (14–16 nm), thick Ag (34–36 nm), IZO (159–161 nm), and DMD (9–11 nm). Combining the two emissive structures with these four top electrodes produced the eight device classes used in this study. The measured QD PL spectrum and wavelength-dependent optical constants were included in the Setfos model.

**Figure 3:**
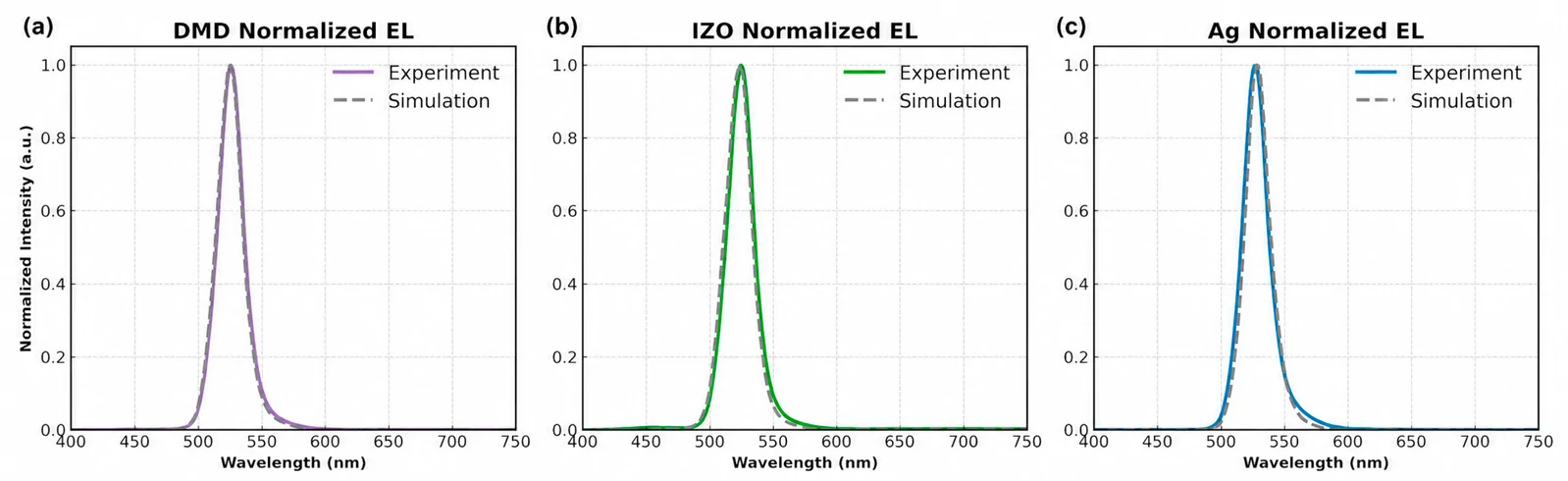
Experimental validation of the optical digital twin using normalized electroluminescence spectra. Measured and simulated EL spectra are compared for QLED devices with (**a**) DMD, (**b**) IZO, and (**c**) Ag top electrodes. The close agreement in peak position and spectral profile confirms that the digital twin can reproduce electrode-dependent microcavity modulation and provides a physically reliable basis for generating training spectra.

Figure 3 compares the simulated electroluminescence spectra with measured spectra from QLED devices using different top electrodes. The DMD, IZO, and Ag devices show different cavity-modulated spectral shapes, and the simulations reproduce the measured peak positions and normalized profiles well. The small remaining differences can arise from fabrication tolerances, uncertainty in the measured optical constants, interfacial roughness, and the finite spectral resolution of the measurement system, none of which is fully represented in the multilayer model. The agreement is sufficient for using the model to generate spectra with controlled thickness variations.

The measured electroluminescence spectra were also included in the training set as experimental samples. These measured spectra were not included in the independent testing set.

To prevent the classifiers from relying mainly on peak wavelength, the optical cavity lengths of all eight device classes were adjusted so that their normal-incidence emission peaks were centered at 528 nm. The top-electrode thickness was then varied by ±1 nm in 0.1 nm steps, giving 21 simulated spectra for each class. These simulated spectra formed the main dataset and were supplemented with measured spectra in the training set. Spectra from the same device structure were assigned the same class label. This design retains cavity-dependent changes in linewidth, curvature, shoulder features, and tail intensity while minimizing peak-position differences. Other functional-layer thicknesses can also affect the optical path length and the EL spectrum, but they were kept fixed here so that the effect of top-electrode thickness could be examined separately.

**Figure 4:**
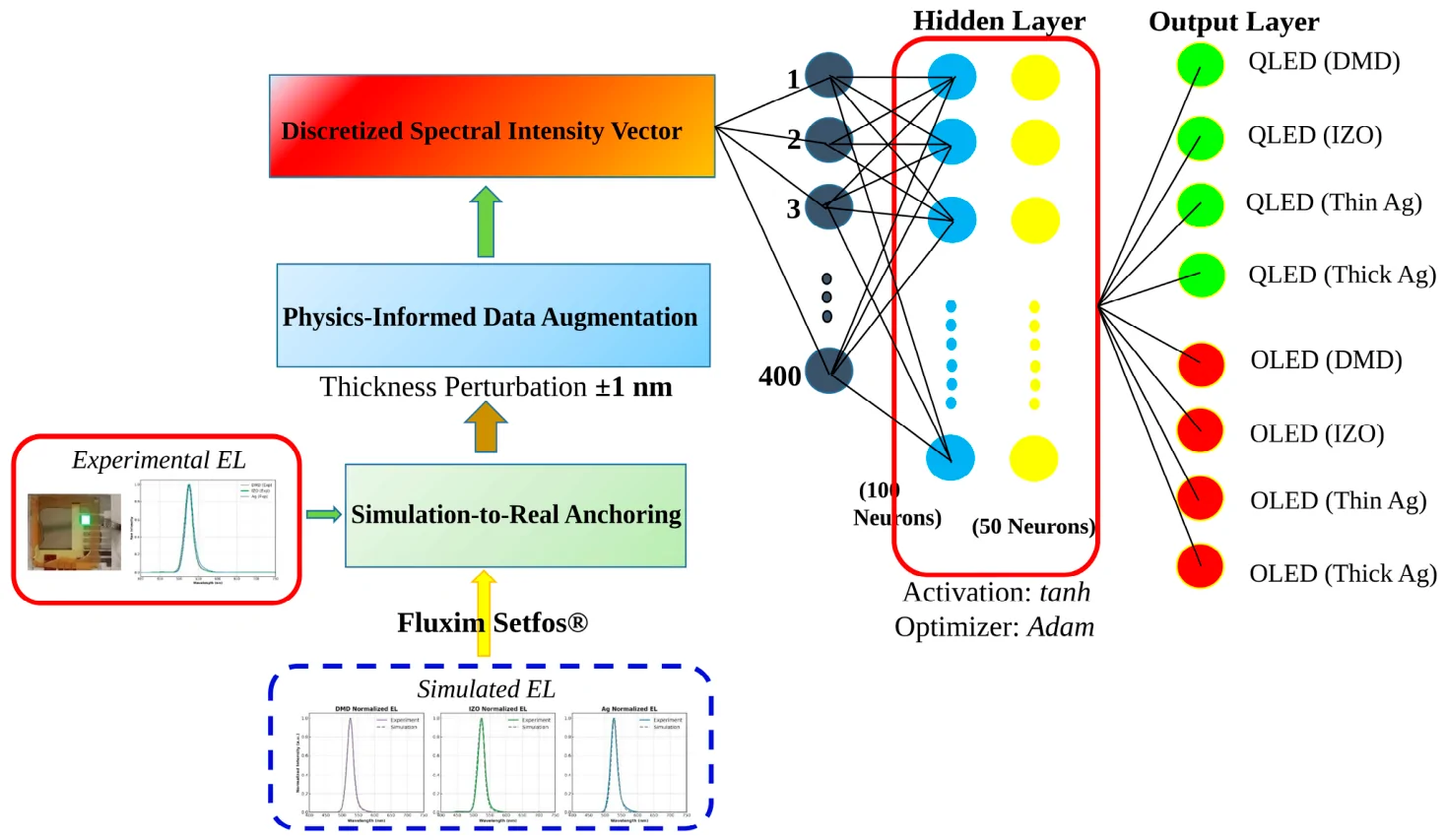
Digital twin-assisted MLP workflow for structural classification. Experimentally parameterized optical simulations generate electroluminescence spectra with ±1 nm top-electrode thickness variations. The spectra are normalized and provided to the two-hidden-layer MLP classifier. Measured EL spectra are used both to check the optical model and as experimental samples in the MLP training dataset. Arrows indicate the workflow between simulation, experimental anchoring, data augmentation, and classification; green and red output nodes represent QLED and OLED classes, respectively.

The electroluminescence spectra from 351 to 750 nm were sampled at 1 nm intervals and normalized to form wavelength-resolved intensity vectors. Four classifiers were evaluated. The simulated dataset was first divided into training and independent testing subsets, with 20% reserved for final testing. PCA was then applied to the Gradient Descent, SVM, and Logistic Regression models for dimensionality reduction. The MLP used the normalized full-spectrum vectors directly, without PCA. It contained two hidden layers with 100 and 50 neurons, used Tanh activation, and had a sigmoid-activated output layer; Adam was used for optimization. Measured spectra were included only in the training data. Five-fold cross-validation was performed within the training set for model evaluation and hyperparameter selection, and the independent test set was not used during these steps. No artificial noise was added to the simulated spectra. The measured spectra included the normal experimental and device-to-device variations present in the measurements. The overall digital twin-assisted classification workflow is shown in Figure 4.

## 3. Results and Discussion

**Figure 5:**
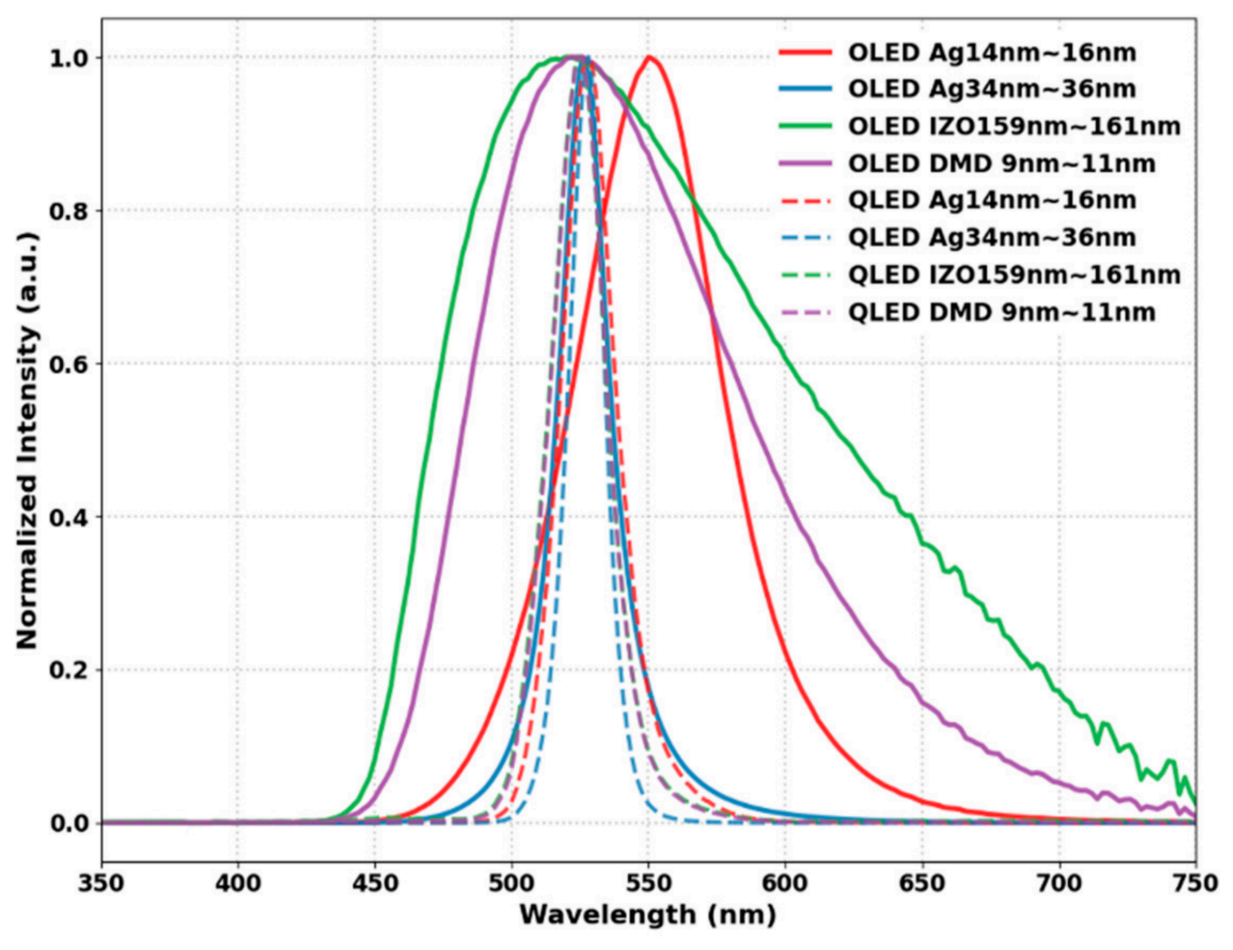
Normal-incidence electroluminescence spectra of OLEDs and QLEDs with different electrode configurations. The thick-Ag OLED shows strong microcavity narrowing and overlaps with the QLED spectra, indicating that peak wavelength and FWHM alone are insufficient for reliable structural identification.

Figure 5 compares the simulated normal-incidence electroluminescence spectra of the eight device classes. OLEDs with IZO and DMD top electrodes show relatively broad emission, whereas the thick-Ag OLED spectrum is strongly narrowed by the microcavity. After the cavity lengths were adjusted, the thick-Ag OLED spectrum overlaps substantially with the QLED spectra around 528 nm. Its linewidth is also similar to that of the QLED spectra. Under these conditions, peak wavelength and FWHM alone cannot reliably distinguish the structures, even though differences remain in the overall spectral shape.

Spectral camouflage in this study does not mean that the spectra are identical at every wavelength. The ambiguity is strongest when the comparison is reduced to descriptors such as peak wavelength and FWHM. The complete spectrum still contains reproducible differences in curvature, linewidth, shoulder structure, and tail intensity. These wavelength-dependent differences are therefore used for the subsequent machine-learning classification.

**Figure 6:**
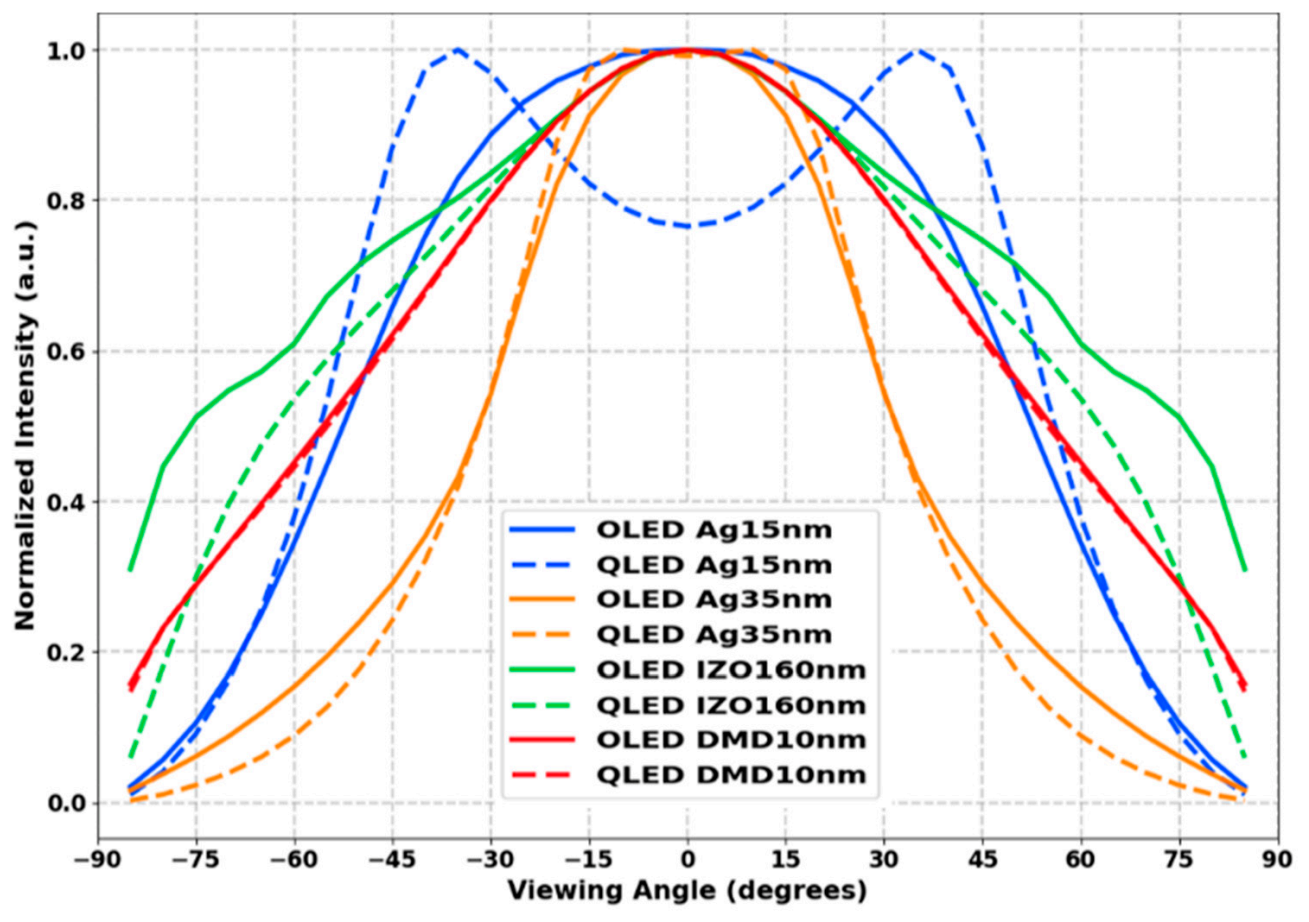
Simulated angular emission characteristics of OLEDs and QLEDs with different electrode configurations. The thick-Ag OLED and QLED structures can show strong spectral overlap at normal incidence but different angular emission because of their different cavity and outcoupling behavior. The angular results are used only for physical interpretation and are not classifier inputs.

Figure 6 shows the simulated angular emission patterns of the devices. Although the QLED and thick-Ag OLED structures can have very similar spectra at normal incidence, their angular emission is different. The QLED devices are consistent with relatively weak-cavity outcoupling from the quantum-dot layer, whereas the thick-Ag OLED shows stronger angle dependence associated with Fabry–Pérot resonance and cavity-mediated outcoupling. The angular results therefore show that the two spectrally similar cases correspond to different optical structures.

The angular simulations were used only to interpret the optical origin of the spectral differences and were not used as classifier inputs. All classification results were obtained from single-shot normal-incidence spectra. The remaining differences at 0°, including linewidth, curvature, shoulder structure, and tail intensity, are difficult to distinguish by visual inspection but are still present in the wavelength-resolved spectra used for classification.

**Figure 7:**
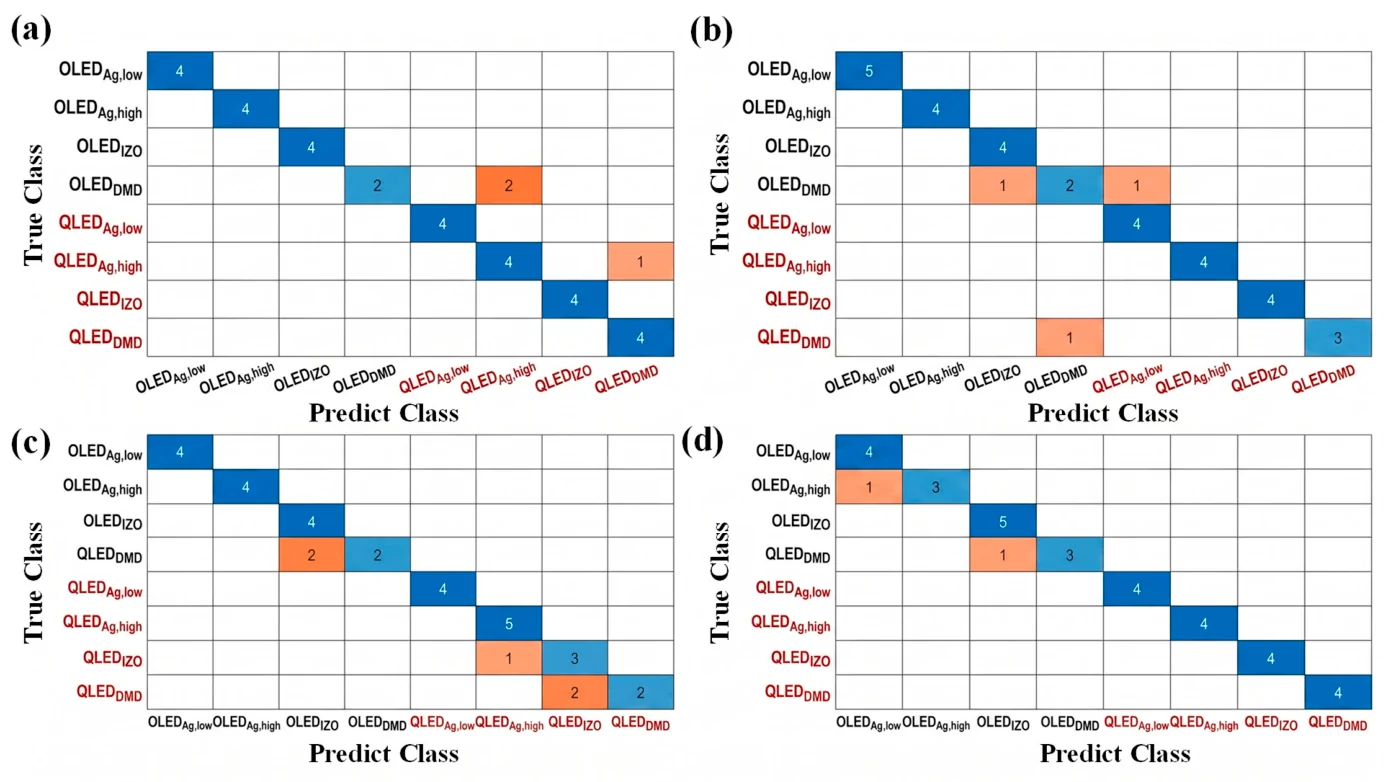
Confusion matrices obtained on the independent testing dataset using (**a**) Gradient Descent, (**b**) SVM, (**c**) Logistic Regression, and (**d**) the Tanh-activated MLP. The corresponding testing accuracies are 90.91%, 90.91%, 84.85%, and 93.94%, respectively. Blue diagonal cells indicate correct classifications, orange off-diagonal cells indicate misclassifications, and the numbers represent sample counts.

Figure 7 presents the confusion matrices for the independent test set using Gradient Descent, SVM, Logistic Regression, and the Tanh-activated MLP. Their testing accuracies were 90.91%, 90.91%, 84.85%, and 93.94%, respectively. All four methods correctly classified most of the eight QLED/OLED structures, but the error patterns differed among the models. Logistic Regression gave the lowest test accuracy, showing that a linear decision boundary did not fully separate the overlapping spectral classes.

The MLP gave the highest testing accuracy, 93.94%, and outperformed the other three classifiers on the independent test set. Its nonlinear mapping appears to capture spectral differences that are not represented well by a linear boundary, including changes in linewidth, curvature, shoulder structure, and tail intensity. The remaining errors also show that the classification problem is not trivial when thickness variations and strongly overlapping spectra are included.

Five-fold cross-validation was also performed on the training data during model evaluation and hyperparameter selection. Table 1 summarizes the selected settings together with the cross-validation and independent test accuracies. The MLP reached 92.03% in five-fold cross-validation and 93.94% on the independent test set. Gradient Descent and SVM each reached 90.91% on the test set, while Logistic Regression reached 84.85%. The lower test accuracy of Logistic Regression shows that the PCA-reduced spectral representation is not fully linearly separable for the overlapping QLED/OLED spectra considered here. By contrast, the MLP operates directly on the normalized full-spectrum vectors and produced the best test result. Its cross-validation and test accuracies were also similar, indicating that the result was not driven by a single train–test split. The independent test samples were drawn from the same ±1 nm manufacturing-tolerance range used to define the digital-twin dataset. The reported accuracy should therefore be interpreted as performance within this controlled process window rather than as evidence of extrapolation to manufacturing conditions outside that range.

## 4. Conclusions

In conclusion, we developed a non-destructive method for distinguishing QLED structures from OLED microcavity structures that produce similar normal-incidence spectra. The optical model was parameterized with measured material data and reproduced the measured QLED spectra for several top-electrode configurations. Strong cavity narrowing in the thick-Ag OLED can produce linewidths comparable to those of QLEDs, making peak wavelength and FWHM insufficient on their own. To make the classification problem less dependent on peak position, the device classes were aligned near 528 nm and top-electrode thickness was varied within ±1 nm. Among the four classifiers, the Tanh-activated MLP gave the best overall result, with 92.03% five-fold cross-validation accuracy and 93.94% independent test accuracy. The results show that differences in the full spectral shape can still be used for structural identification under spectral camouflage. The method may therefore be useful as a rotation-free optical screening approach for microcavity display structures.

## Figures and Tables

**Table 1:** Summary of the key hyperparameter settings, cross-validation accuracies, and testing accuracies of the four machine-learning models for QLED/OLED spectral classification.

Model	Key Hyperparameters	Test Accuracy	5-Fold CV Accuracy
Gradient Descent	PCA preprocessing; η = 0.26827	90.91%	82.22%
SVM	PCA preprocessing; C = 70	90.91%	83.70%
Logistic Regression	PC = 9, C = 93	84.85%	91.85%
Tanh MLP	Hidden layers = (100, 50); Tanh activation; Sigmoid output; Adam; No PCA	93.94%	92.03%

## Data Availability

The raw data supporting the conclusions of this article will be made available by the authors on request.
